# The 2026 ISCB Overton Prize Award—Dr Marinka Zitnik

**DOI:** 10.1093/bioinformatics/btag282

**Published:** 2026-07-07

**Authors:** Mallory L Wiper

**Affiliations:** The International Society for Computational Biology, 525K East Market Street, RM 330, Leesburg, VA 20176, United States


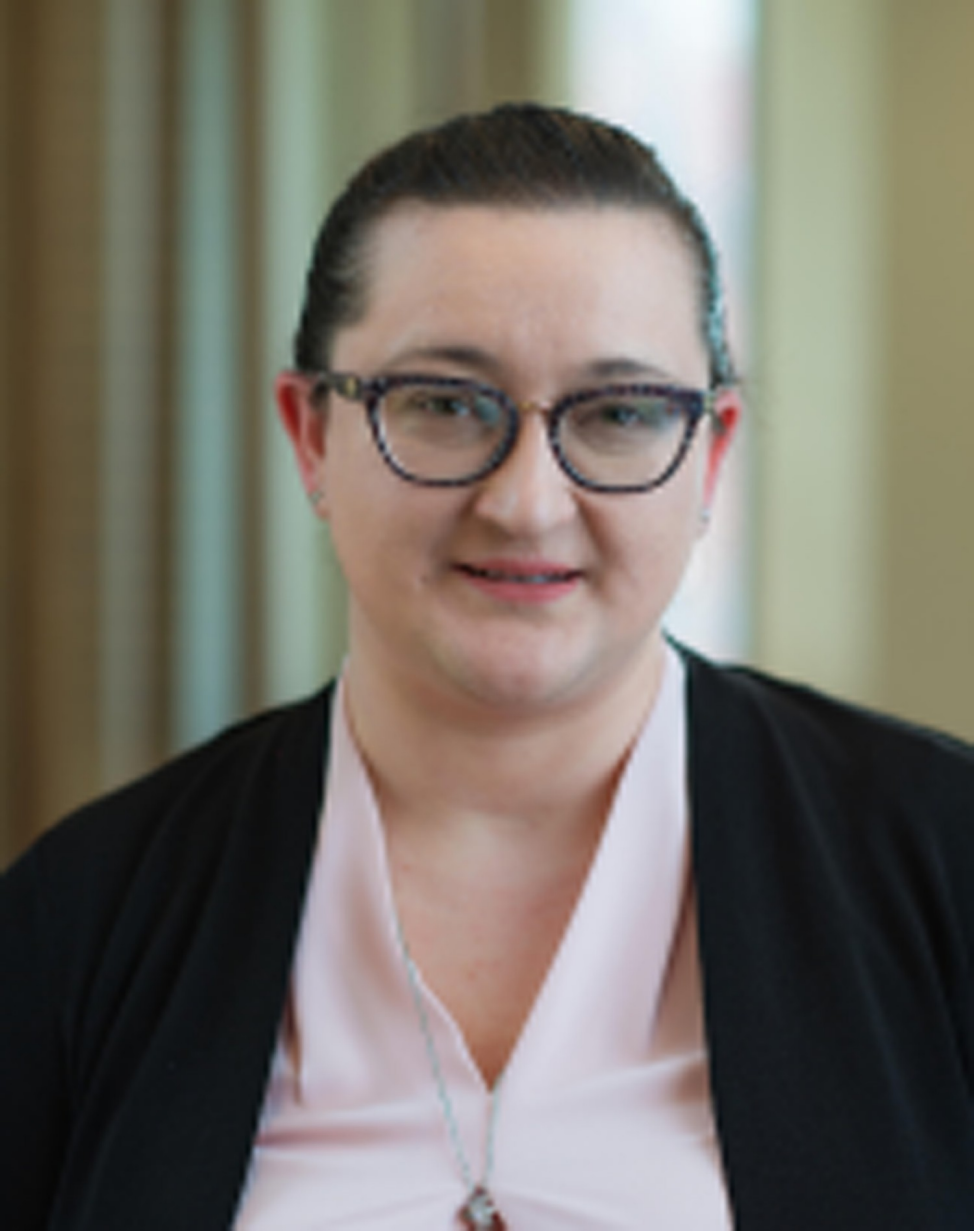


The International Society for Computational Biology (ISCB) is pleased to present this year’s Overton Prize to Dr Marinka Zitnik, in recognition of her significant contributions to computational biology through the development of foundational AI methods to accelerate biomedical discovery. Dr Zitnik will receive her award at the 34th Intelligent Systems for Molecular Biology (ISMB) conference in Washington, D.C., this July.

## 1 A change of course

From a young age, Dr Marinka Zitnik had what she called “a very systematic way of problem solving,” rooted in her love of math, logic, and computer science—topics she pursued not just at school but at home too, where her hobbies included playing chess and solving logic puzzles with her parents.

Though she was interested in math and logic, Zitnik’s career aspiration was to become a medical doctor. However, this aspiration changed when she learned about the emerging field of computational biology, a field combining computation with biological and medical data. Excited by the prospect of building tools that generated real hypotheses for wet lab researchers and that had a tangible impact beyond her own work, she opted to pursue computational biology as a way to combine her skills in math and computer science to systematically solve real-world problems in biology and medicine.

It was Dr Blaz Zupan’s introductory course on computational biology that truly set her on her way. Soon after taking the course, she was working in Zupan’s lab as an undergraduate researcher, where she had the opportunity to collaborate with scientists at the Baylor College of Medicine to identify key genes driving resistance responses to bacterial infection in social amoebas. This collaboration allowed her to see the larger impact of the algorithms and models developed by computational biologists, cementing her desire to pursue computational biology as her focal field of research.

## 2 Formative influences

Two mentors stand out in Zitnik’s mind as being most influential to her academic career.

First was Blaz Zupan, responsible for introducing Zitnik to computational biology. What’s more, by bringing her onto a project to collaborate with researchers from Baylor College, Zupan provided Zitnik with her first opportunity to see how computational methods could be applied to questions in other fields.

Zitnik also named her postdoc advisor at Stanford, Jure Leskovec, as a highly influential mentor. Something she remembers fondly about Leskovec is his can-do attitude and unwavering support of her research ideas—especially since no one else in his lab at that time was working on biological questions. Under Leskovec, Zitnik learned how best to critically assess cutting-edge AI models developed for non-biological data and, through consideration of necessary additional features, properly adapt them to a biological context.

Beyond advising Zitnik in technical skills, Leskovec provided invaluable guidance on making the transition from postdoc to independent researcher. He advised her on what steps to take to set up her own lab and how best to help junior researchers become great scientists. Zitnik said that Leskovec taught her “how to become an effective mentor, an effective teacher, and an effective communicator in science”; all skills she continues to draw on as a mentor and PI in her own right.

## 3 Cheerleader, collaborator, mentor

The support and openness to new ideas that Zitnik saw modeled by her mentors have shaped her own mentoring philosophy. Above all, Zitnik describes herself as a cheerleader for her students. A part of this, too, is helping her students navigate the research process, steering them away from common pitfalls and mistakes while encouraging their pursuit of ambitious ideas.

As their cheerleader, Zitnik sees it as her responsibility to create opportunities for her students, whether that’s through facilitating research collaborations, supporting their applications for fellowships and internships, or helping them design research projects that best align with their strengths. Her goal is to foster her students’ growth as independent researchers, helping to position them as stand-out scientists who consistently make unique contributions to their areas of research.

Zitnik also encourages an environment of collaboration within her research group, where everyone is encouraged to share ideas, engage in discussion, and contribute to the critical assessment of what projects to pursue. Rather than forcing a research direction, her group works together to identify projects and directions that will have the most meaningful impact.

## 4 Curiosity and the PI perspective

Relative to her time as a student, the questions she’s asking with her research pursuits might be different, but Zitnik’s core motivation for research—her innate curiosity and excitement about discovery—hasn’t changed. She remains driven by the magical moment of discovery when a researcher uncovers something new and, for a brief moment, is the only person who knows it.

What *has* changed for Zitnik’s research is that, as a PI, there’s a greater scope and responsibility to consider. Instead of focusing on individual projects, she now must think about the broader direction of her lab, identifying research directions that will be relevant in at least the next five years—which can be difficult, especially in the fast-moving field of AI research. Zitnik works to balance long-term vision with short-term actionable plans for projects within her lab, a forward-looking perspective she didn’t always need to consider.

## 5 Unexpected advances and new directions

A recent shift in Zitnik’s research has been driven by the advances of AI scientists—large language model-powered systems designed to collaborate with human researchers, biologists, and clinicians. Based on these AI scientist models, Ayush Nori, a post-graduate student in Zitnik’s lab, developed an AI model called Proton to generate predictions for three neurological diseases: Parkinson’s disease, bipolar disorder, and Alzheimer’s disease. The predictions were validated through experimentation across molecular, organoid, and clinical systems, resulting in changes expressing functional improvement.

Current research in Zitnik’s lab is continuing to explore the utility of AI scientists as collaborators who can use scientific tools, query experimental and clinical data, and generate and refine hypotheses. Her lab has been dialing in on therapeutic reasoning, with most of the current projects focused on developing AI models that can help with drug discovery and repurposing, motivated by the number of rare diseases that currently lack effective treatments. She is also keenly interested in using AI to model molecules and molecular interactions with the goal of accelerating the nomination of new targets and the design of chemical compounds against them.

For Zitnik, the implications are clear: “In addition to disease diagnosis, disease treatment is where AI models could provide tremendous value to help humanity.”

## 6 Reflections on the Overton Prize Award

Zitnik said she’s incredibly honored to be the 2026 Overton Prize winner, joining a community of fantastic researchers she has long admired. She also emphasized that her receipt of this award is not an individual achievement, crediting her mentors for their support, guidance, and openness to new research directions. She expressed equal gratitude for her students, whose work, creativity, and belief in and support of her research vision—especially during her early years as a PI—have been instrumental to her contributions to the computational biology community.

